# Nitro-Hydrochloric Acid in Dentistry

**Published:** 1899-05

**Authors:** 


Nitro-Hydrochloric Acid in Dentistry.—If it is possible to exclude air from a tooth cavity, or root canal, after having treated the same with nitro-hydrochloric acid then is it possible to obtain complete and permanent asepsis.

After treating a cavity with this acid, nitrous acid gas and chlorine gas are apt to be formed, both of which are undoubtedly germicidal, and consequently calculated to prevent further decay. Continued experimentation is, however, necessary in order to learn whether the action of the acid on the albuminous matter of the teeth is not so severe as to offset the aseptic benefits secured. Acid albumin would no doubt be formed and certain coagulations result. Clinical results alone can prove the efficacy of the treatment.

